# Non-neuronal evoked and spontaneous hemodynamic changes in the anterior temporal region of the human head may lead to misinterpretations of functional near-infrared spectroscopy signals

**DOI:** 10.1117/1.NPh.5.1.011002

**Published:** 2017-08-22

**Authors:** Guilherne Augusto Zimeo Morais, Felix Scholkmann, Joana Bisol Balardin, Rogério Akira Furucho, Renan Costa Vieira de Paula, Claudinei Eduardo Biazoli, João Ricardo Sato

**Affiliations:** a NIRx Medizintechnik GmbH, Berlin, Germany; b University of Zurich, University Hospital Zurich, Biomedical Optics Research Laboratory, Department of Neonatology, Zurich, Switzerland; c Universidade Federal do ABC, Center for Mathematics Computing and Cognition, São Bernardo do Campo, Brazil; d Instituto do Cérebro, Hospital Israelita Albert Einstein, São Paulo, Brazil

**Keywords:** functional near-infrared spectroscopy, optical neuroimaging, superficial temporal vessels, scalp blood flow, temporalis muscle, extracerebral signal contamination, temporal lobe

## Abstract

Several functional near-infrared spectroscopy (fNIRS) studies report their findings based on changes of a single chromophore, usually concentration changes of oxygenated hemoglobin ([$O_{2}\mathrm{Hb}$]) or deoxygenated hemoglobin (HHb). However, influence of physiological actions may differ depending on which element is considered and the assumption that the chosen measure correlates with the neural response of interest might not hold. By assessing the correlation between [$O_{2}\mathrm{Hb}$] and [HHb] in task-evoked activity as well as resting-state data, we identified a spatial dependency of non-neuronal hemodynamic changes in the anterior temporal region of the human head. Our findings support the importance of reporting and discussing fNIRS outcomes obtained with both chromophores ([$O_{2}\mathrm{Hb}$] and [HHb]), in particular, for studies concerning the anterior temporal region of the human head. This practice should help to achieve a physiologically correct interpretation of the results when no measurements with short-distance channels are available while employing continuous-wave fNIRS systems.

## 1 Introduction

Functional near-infrared spectroscopy (fNIRS) is an optical neuroimaging technique enabling to quantify changes in hemodynamics and oxygenation within the intracerebral and extracerebral tissue layers of the human head based on the absorption of near-infrared light by the oxygenated ($O_{2}\mathrm{Hb}$) and deoxygenated (HHb) forms of hemoglobin.^1^^,^^2^ Similar to functional magnetic resonance imaging (fMRI) and its blood-oxygen-level-dependent (BOLD) signal, the inference on neural activation relies on the principle of neurovascular coupling and thus a close relation with changes in cerebral blood flow.^3^ However, while traditional fMRI inference is based solely on the BOLD signal, fNIRS has expanded the sensitivity to intrinsic chromophores, including $O_{2}\mathrm{Hb}$, HHb, and the oxidized form of the cytochrome c-oxidase (oxCCO),^4^ in addition to further dependent quantities, including total hemoglobin, tissue oxygen saturation, and $O_{2}\mathrm{Hb}$ exchange efficiency.

Based on the modified Beer–Lambert law,^5^ the number of different intrinsic chromophores that can be measured by fNIRS systems is mathematically limited by the number of wavelengths employed for the measurement. Different studies have shown that for a proper measure of $O_{2}\mathrm{Hb}$ and HHb, two wavelengths are general enough to yield meaningful measures when properly chosen.^6^^–^^8^ Although the great majority of commercially available fNIRS systems employs at least two wavelengths for the measurement,^1^ many reports are still based on a single chromophore, usually $O_{2}\mathrm{Hb}$ or HHb, even though studies have shown that these measures are individually influenced by the underlying physiology.^9^^–^^11^ Activation inference based on neurovascular coupling foresees an increase of oxygen metabolism followed by an outweighed increase of cerebral regional blood flow, thus resulting in an increase in the concentration of $O_{2}\mathrm{Hb}$ ([$O_{2}\mathrm{Hb}$]) expected to be accompanied by a decrease in concentration of HHb ([HHb]).^12^ Thus, the interpretation of the results should be based on both chromophores.

Report findings based on a single chromophore assume that the chosen measure correlates with the neural response of interest. However, the observation of an increase of [$O_{2}\mathrm{Hb}$] alone may not be enough to make proper inferences about the underlying neural activity, as an accompanying increase of [HHb] could be an indicator of hemodynamic changes not evoked by neural activity, happening in the intracerebral and/or extracerebral tissues.^13^^,^^14^ In fact, Tachtsidis and Scholkmann^15^ recently summarized different physiological sources that may lead to false positives and false negatives in fNIRS measures and the reality is that the fNIRS signal assumed to always be neuronally evoked may be present in many other physiological components. These include changes in partial pressure of carbon dioxide in the arterial blood (${\mathrm{PaCO}}_{2}$),^16^^,^^17^ blood pressure,^18^^,^^19^ and activity of the sympathetic nervous system.^19^^,^^20^ As shown in these studies, the physiological confounds may lead to non-neuronally evoked responses, therefore, corresponding result interpretations should be done carefully because the hemodynamic responses measured are not due to neurovascular coupling.

In the present study, we report observations that identify a spatial dependency of non-neuronal hemodynamic changes to be of significance for fNIRS studies: hemodynamics on the anterior temporal region, which might be caused by “head muscles activity,” as also recently reported by Volkening et al.^21^ Based on the expected negative correlation between [$O_{2}\mathrm{Hb}$] and [HHb] for task-related responses, we observed that the anterior temporal region may be especially susceptible to this behavior. In addition, we demonstrate that the negative [$O_{2}\mathrm{Hb}$] to [HHb] correlation is sustained for resting-state spontaneous fluctuations for all cortical regions on the left hemisphere but not for the anterior temporal area.

## 2 Materials and Methods

### 2.1 Data

**Fig. 1:**
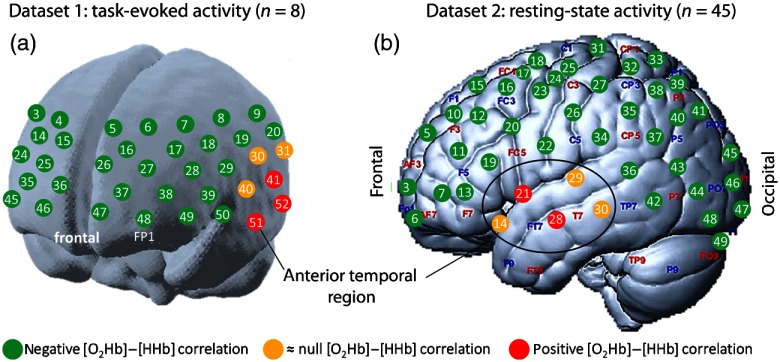
Distribution of the source and detector locations for the task-related (a, created based on Refs. 22 and 23) and resting-state related data (b). Channels in green presented negative [$O_{2}\mathrm{Hb}$] to [HHb] correlation, the channels in red yielded a positive correlation, and the channel in yellow presented median correlation close to zero (within the range limited by −0.1 and 0.1). The correlation results shown in this figure correspond to group-level results, as described in Sec. 3.

In this study, we have assessed the correlation between [$O_{2}\mathrm{Hb}$] and [HHb] in two different datasets of experiments involving the measurement of (i) task-related and (ii) resting-state brain activities. The materials and methods corresponding to each dataset are described in detail in the following sections. The spatial distribution of channels used to obtain each set of data is shown in Fig. 1.

#### 2.1.1 Dataset 1: task-evoked activity

The first dataset used for the present study is publicly available^24^ and was originally collected to evaluate the task-evoked responses related to a simple mental arithmetic paradigm. Details on the original experimental design and results obtained can be found in the corresponding report.^22^

The dataset has been acquired with a continuous-wave NIRS system (ETG-4000, Hitachi Medical Corporation, Osaka, Japan) that employs two different wavelengths (695 and 830 nm) for intensity data acquisition and calculation of [$O_{2}\mathrm{Hb}$], [HHb], and total hemoglobin ($\left[\mathrm{tHb}]=[O_{2}\mathrm{Hb}]+[\mathrm{HHb}\right]$) concentration changes via the modified Beer–Lambert law.^5^

For the measurement, 17 light sources and 16 detectors have been arranged in a 3×11 grid covering frontal and temporal regions of the cortex, as shown in Fig. 1(a), yielding 52 channels in total. The sampling frequency was set to 10 Hz and the source–detector separation was fixed to 30 mm. The lowest line of sources and detectors was placed along the FP1–FP2 line of the 10 to 20 international system^25^ with channel 48 exactly at FP1 position.

The experimental design consisted of six blocks (per run) of a mental arithmetic task performed during 12 s followed by a resting period of 28 s. Participants were instructed to mentally subtract a one-digit number from a two-digit number as quickly as possible within the block period. The first subtraction was presented as a visual cue on the screen and the following subtractions should be based on the two-digit result and the initial one-digit number presented (e.g., cue: 97−4→substraction to be performed: 97−4=93, 93−4=89, 89−4=85, etc.).

The dataset downloaded consists of unfiltered concentration changes obtained from eight subjects (mean age: 26.0 years, standard deviation: 2.8 years, males: 3) separated in different runs. Subjects 01 to 03 contain three runs, whereas the datasets for subjects 05 to 08 have four runs. Data are provided with the scale unit of [mM mm] representing the molar concentration [mM=mmol/L] multiplied by the unknown pathlength of each wavelength [mm]. Information on the trigger onsets of each trial of task and rest are also provided. Before proceeding to data analysis based on correlation (as described in Sec. 2.2.1), we applied a bandpass filter of [0.01 to 0.20] Hz (filter type: linear-phase FIR, roll-off width: 15%).

#### 2.1.2 Dataset 2: resting-state activity

The second dataset was collected at the Universidade Federal do ABC following approval by the local Ethics Committee and upon written informed consent provided by all participants.

**Fig. 2:**
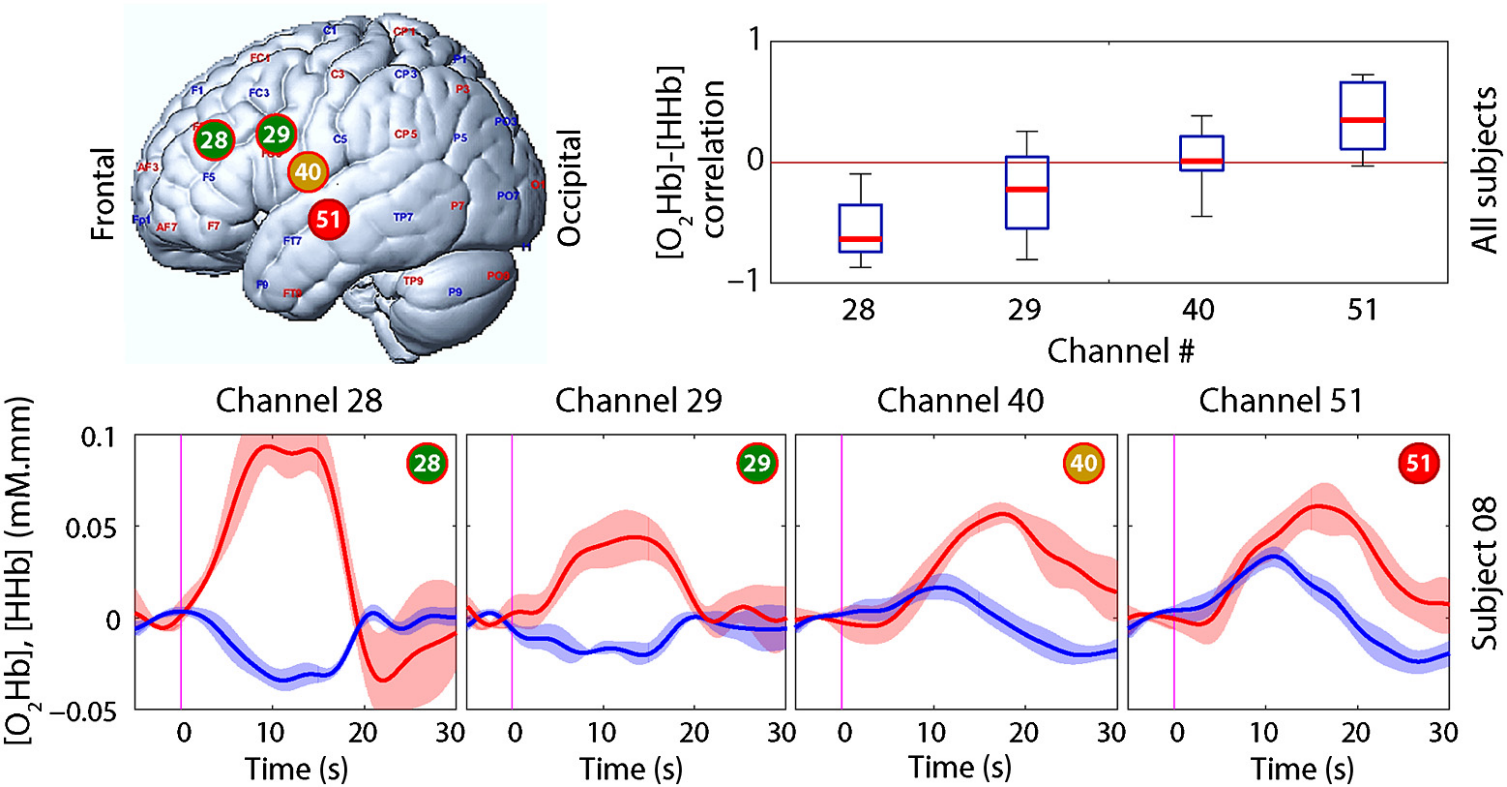
Task-evoked results for four illustrative channels with color correspondence to the spatial distribution in Fig. 1(a). Boxplots with correlation results over all subjects are shown on the top. Block average results (over runs) of a representative subject (#08) are depicted on bottom. [$O_{2}\mathrm{Hb}$] time series are plotted as red and [HHb] as blue signals. The shaded area represents the standard deviation.

A continuous-wave NIRS system (NIRScout, NIRx Medical Technologies, Glen Head, New York) that uses two different wavelengths (760 and 850 nm) was employed. We used 16 light sources and 16 detectors to cover most of the left hemisphere of the participants, as shown in Fig. 2(a). This layout resulted in 49 channels measured with a sampling frequency of 3.91 Hz. Sources and detectors positions were based on the 10-5 international system^25^ and the source–detector separations ranged from 20 (channels 17 and 24) to 30 mm (all other channels). The upper separation limit was achieved with the aid of stabilizing links provided by the manufacturer and placed for all channels between their forming source and detector positions.

Ten different regions of interest were visually defined in respect to underlying anatomical regions (Table 1). To retrieve the channel positions, we considered the MNI coordinates of the 10-5 international system positions as provided by Jurcak et al.^25^ We imported them into the SPM for fNIRS toolbox^26^ to obtain the channels distribution, based on which we generated the montage representation that is shown in Fig. 1(b).

**Table 1:** Defined regions of interest for resting-state data and corresponding channels. Individual channel positions are shown in Fig. 1(a).

Region of interest	Channels
Frontal anterior	1, 2, 3, 4, 6, 8
Frontal superior	5, 9, 10, 15, 16
Frontal inferior	7, 11, 12, 13, 19, 20
**Temporal anterior**	**14, 21, 28, 29, 30**
Temporal posterior	36, 42, 43, 44
Premotor	17, 18, 22, 23
Motor	24, 25, 26, 31
Parietal inferior	34, 35, 37, 38, 40
Parietal superior	27, 32, 33, 39, 41
Occipital	45, 46, 47, 48, 49

Resting-state time series were collected from 45 participants (mean age: 24.2 years, standard deviation: 3.8 years, males: 39) during ∼5  min. For consistency purposes, data from all participants were truncated during offline processing from the first data point to the 1172nd, therefore, resulting in exactly 5 min long time series. Prior to data acquisition, subjects were instructed to keep their eyes opened and not to perform structured thought processes during the measurement.

The coefficients of variation (CVs) of the truncated intensity data were then calculated for each channel and from each subject according to CV(%)=100×standard deviation (data)/mean (data).

The CV has been used as a metric for signal quality control. All channels with a CV>7.5% have been excluded from the data analysis because they contained unphysiological noise. Data collected from 38 to 45 subjects were considered per each channel. In the supplementary material,^27^ we have included a table with the number of subjects considered per channel of interest.

After performing a signal quality check, intensity data have been bandpass filtered within the range (0.01 to 0.20) Hz (filter type: linear-phase FIR, roll-off width: 15%). The lower cutoff frequency was set to remove very low frequency oscillations and the higher to eliminate both respiratory and cardiac components of the signal.

The filtered data were then converted to optical density data and then to ([$O_{2}\mathrm{Hb}$] and [HHb]) changes based on the modified Beer–Lambert law.^5^ The extinction coefficients used for 760 nm were 1.4866 ([$O_{2}\mathrm{Hb}$]) and 3.8437 ([HHb]), whereas for 850 nm they were 2.5264 ([$O_{2}\mathrm{Hb}$]) and 1.7986 ([HHb]) in units of [(1/cm)/(mmol/L)]. The different pathlength factor (DPF)^28^ was calculated based on the general equation proposed by Scholkmann and Wolf^29^ considering a mean age of 24 years for all subjects and yielding 6.12 for 760 nm and 5.06 for 850 nm. Finally, the baseline was defined as the mean of the whole time series (5 min).

### 2.2 Data Analysis

As previously mentioned, both datasets underwent analysis with the goal to assess the correlation between [$O_{2}\mathrm{Hb}$] and [HHb]. In addition, we employed further metrics to better quantify and understand the relation of these results with other behaviors of interest. For the dataset with task-evoked data, we also evaluated the grand average of the blood volume pulse amplitude (BVPA); and for the resting-state, channel-wise correlation for each chromophore was calculated for all channels as seeds.

#### 2.2.1 [O_2_Hb] to [HHb] correlation

To quantify the correlation between [$O_{2}\mathrm{Hb}$] and [HHb], the Spearman’s correlation coefficient was chosen because this parameter does not expect that the signals have a linear relationship with each other, and it is also more robust against outliers (e.g., motion artifacts within the window of interest).

For the task-related data, we calculated the Spearman’s correlation for each mental arithmetic trial within a 30-s window following the onset time to accommodate the complete hemodynamic response until the signal returned to its baseline. We computed the median of the correlation results over trials to obtain the correlation per run. Similarly, the correlation for a given subject was obtained with the median over runs. This resulted in a matrix with eight rows (subjects) and 52 columns (channels).

For the resting-state dataset, as there is no defined task-window as a reference to compute the correlation, we rather calculated it based on a 20 s sliding window. The length of the window has been chosen arbitrarily. The correlation for a given subject was based on the median over all windows within the time series. Similarly, this resulted in a matrix with 45 rows (subjects) and 49 columns (channels). However, in this case, entries corresponding to the excluded channels were replaced with not-a-number (NaN) values to be ignored in the group-level correlation and distribution results. The procedure of replacing results obtained from excluded channels with missing data was done to avoid bias of nonsatisfactory signal quality in the results and achieved using MATLAB functions “nanmedian” and “boxplot,” which ignore NaN values.

The matrix of correlation results for both task-related and resting-state datasets was then used to generate boxplots for each channel to better illustrate the distribution over subjects. In addition, we computed the median correlation over subjects.

#### 2.2.2 Blood volume pulse amplitude

Concerning the hemodynamics related to the task-related dataset, we hypothesized that a further metric to evaluate the influence of systemic changes in each channel would be the BVPA. The blood volume pulsation due to the periodic heart activity is generally present in fNIRS signals.^30^^,^^31^

First, we considered the unfiltered concentration changes and applied a different bandpass filter of [0.50 to 2.50] Hz (filter type: linear-phase FIR and roll-off width: 15%) to extract the blood volume pulsation from the signals. Then, we computed the BVPA as the difference between the upper and lower envelopes of the heartbeat oscillation from [tHb]. Upper and lower envelopes have been computed based on the peak-detection algorithm described by Scholkmann et al.^32^

As our hypothesis is based on the BVPA response as evoked by the task, we computed block averages of the BVPA responses within the window [−5, 30] s, in which 0 s corresponds to the mental arithmetic trial onset. The window upper limit corresponds to that used for the correlation computation (see Sec. 2.2.1) and the lower limit is to allow for 5 s prior to the onset to be counted as a baseline. The baseline mean was subtracted from each data point within the following 30 s of response block to properly normalize the blocks’ prior averaging.

Similar as for the correlation calculation, we computed the mean of block averages results over runs and then over subjects. We preferred to use the “mean” function (instead of “median”) to preserve the continuity of the signal within the response window for the grand average.

Finally, we computed further metrics of interest based on the grand average results, now within the window [0, 30] s, as follows: (a) peak-to-peak, (b) mean, (c) variance, and (d) median absolute deviation. These were calculated to have a single representative value for the systemic influences in each channel to be related to the correlation results obtained for the same channel, as shown in Sec. 3.1.2.

#### 2.2.3 Channel-wise correlation

For the resting-state dataset, we computed the channel-wise correlation as a metric to assess the global influence of expected spontaneous vascular and metabolic fluctuations that are present in both task and resting states.^33^^,^^34^ As the primary analysis goal was to evaluate the presence of global fluctuations, we did not apply any further correction (e.g., principal component analysis^35^^,^^36^).

Similar to [$O_{2}\mathrm{Hb}$] to [HHb] correlation analysis (Sec. 2.2.1), we used a sliding window of 20-s length here to calculate the Spearman’s correlation between all channels for each chromosphere, ([$O_{2}\mathrm{Hb}$], [HHb], and [tHb]), for each subject. After that, we computed the median correlation value over all subjects. This procedure resulted in a correlation matrix of 49 rows and 49 columns (49 is the number of measured channels) for each chromophore. An (X,Y) entry in the matrix, therefore, represents the median correlation between channel X and channel Y for a given chromophore over all subjects.

The global influence of spontaneous signals’ fluctuations was assessed with the median correlation value over all channels, yielding a vector of length 49 for each chromophore. An entry is a single representative value on the correlation between a given channel and all other measured channels.

## 3 Results

### 3.1 Dataset 1: Task-Evoked Activity

#### 3.1.1 At the anterior temporal region, the [O_2_Hb] response is positively correlated with the [HHb] response

According to the correlation analysis described in Sec. 2.2.1, we obtained results that demonstrate a consistent positive correlation between [$O_{2}\mathrm{Hb}$] and [HHb] on channels over the anterior temporal region in both hemispheres. The results for all channels are available in the supplementary material.^27^

Figure 2 shows the correlation distribution of four particular channels (28, 29, 40, and 51) that are highlighted in Fig. 1 as green, yellow, and red circles. Boxplots and their central marks gradually shift toward positive correlation values while the measurement channels get closer to the anterior temporal area. In particular, channel 51 (red) presents not only the median value, but also shows the first quartile in the positive area.

The block averages also illustrate the phenomenon observed with the boxplots distribution, i.e., how the task-related responses gradually shift from the expected [$O_{2}\mathrm{Hb}$] increase and [HHb] decrease to a concomitant increase of both chromophores over the anterior temporal area.

#### 3.1.2 At the anterior temporal region, the [O_2_Hb] to [HHb] correlation strength is correlated with the blood volume pulse amplitude

As outlined in Sec. 2.2.2, we have hypothesized the task-evoked response of the BVPA to be an indicator related to the influence of systemic changes in that channel. Therefore, we calculated the grand average results of BVPA for each channel available and considered four different metrics to explore their relation to the previously obtained [$O_{2}\mathrm{Hb}$] to [HHb] correlation over all subjects.

**Fig. 3:**
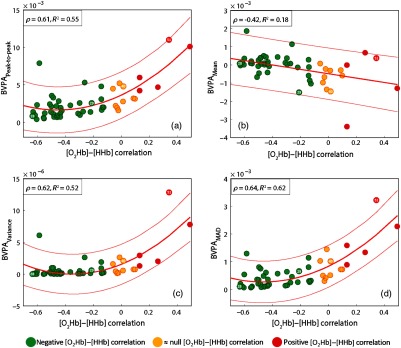
Scatter plots of different metrics of the BVPA (y-axis) with respect to the [$O_{2}\mathrm{Hb}$] to [HHb] correlation (x-axis) for task-evoked data. From left to right and then top to bottom, the BVPA metrics are: (a) peak-to-peak, (b) mean, (c) variance, and (d) median absolute deviation (mad). For each metric, a polynomial fit has been applied to the data (red), whereas the orange curves represent the 95% confidence interval. A linear fit was empirically found to better fit the mean, whereas the quadratic fit for all the other metrics. Each graph presents the correlation (ρ) between the variables as well as the adjusted coefficient of determination ($R^{2}$). Colored points (green, yellow, and red) relate to color-coded channels in Fig. 1(a). Channels highlighted in Fig. 2 are marked with a white number (28, 29, 40, and 51).

From all four metrics explored in Fig. 3, the quadratic fit for peak-to-peak, variance, and median absolute deviation has resulted in the greatest correlation (ρ) values as well as adjusted coefficient of determination ($R^{2}$). Although the mean scatter plot presents a relatively high absolute ρ (0.42), the $R^{2}$ is rather low due to the greater distance between fitted line and spread points.

Interestingly, the green points (channels 28 and 29) both presented low [$O_{2}\mathrm{Hb}$] to [HHb] correlation and low BVPA results [metrics (a), (c), and (d)], whereas the yellow (channel 40) lies on a transition portion and the red (channel 51) presents relatively high values for both correlation and BVPA results [(a), (c), and (d)].

### 3.2 Dataset 2: Resting-State Activity

#### 3.2.1 At the anterior temporal region, the [O_2_Hb] oscillations are positively correlated with the [HHb] ones

As described in Sec. (2.2.1), for the resting-state dataset, we have obtained a correlation matrix with 45 rows (subjects) and 49 columns (channels). To avoid plotting all 49 channels individually, and as these results can be found in the supplementary material,^27^ we preferred to group them in 10 different regions of interest, which consisted of groups from four to six channels (Table 1). Therefore, before generating the boxplots results with the distribution over all subjects, we first calculated the mean results within each region of interest.

**Fig. 4:**
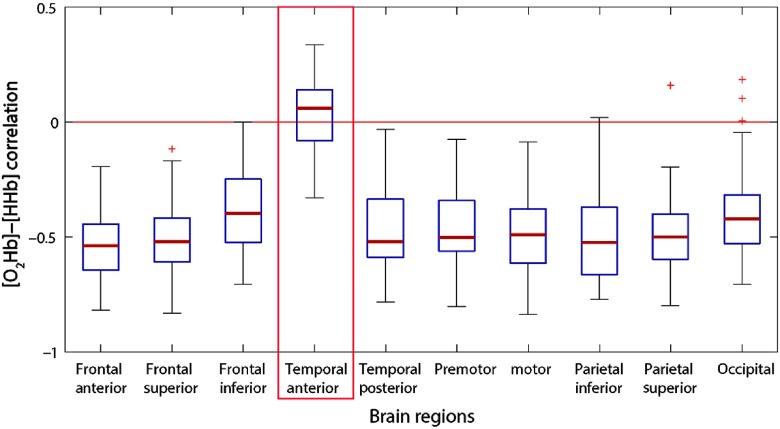
Boxplots illustrating the [$O_{2}\mathrm{Hb}$] to [HHb] resting-state correlation results over all subjects for each region of interest according to the underlying anatomical regions. The red box highlights the anterior temporal region (channels 14, 21, 28, 29, and 30).

From the results presented in Fig. 4, the most important remark is related to the central mark of each boxplot (median value) for each region of interest. While the median result for the anterior temporal region represents a positive correlation, the central mark of all other regions of interest is around −0.5 instead, whereas their third quartile is considerably below the null correlation, too.

#### 3.2.2 At the anterior temporal region, the [O_2_Hb] to [HHb] correlation strength depends on the channel-wise correlation strength of [O_2_Hb], [HHb], and [tHb]

According to the methods described in Sec. 2.2.3, for the resting-state data, we have also assessed the influence of global components on each channel and explored its relation to the previously obtained [$O_{2}\mathrm{Hb}$] to [HHb] correlation.

**Fig. 5:**
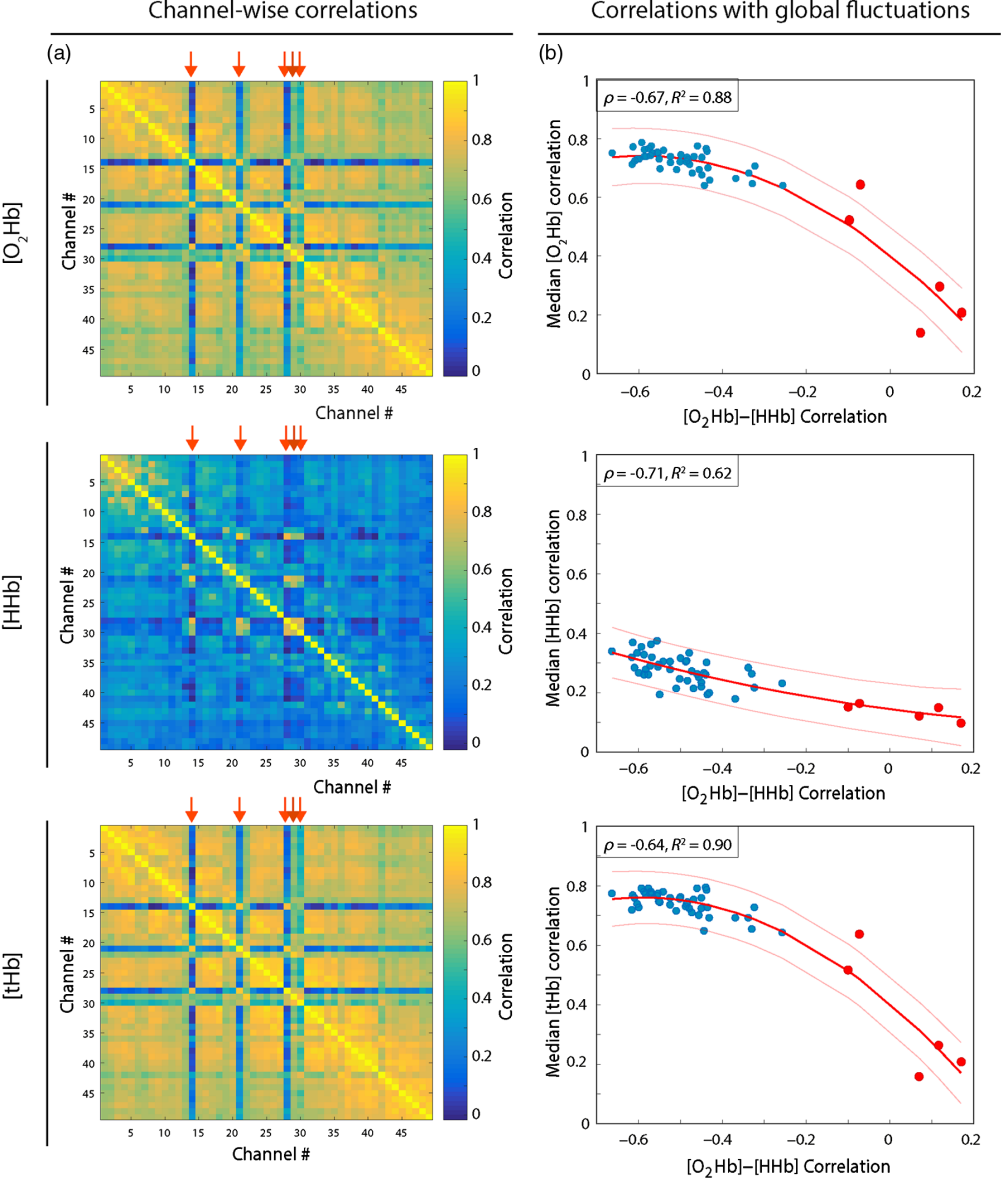
(a) The median channel-wise correlation over all subjects (resting-state data) is depicted with scaled colors for different chromophores. Orange arrows have been added on top of each image to indicate the channels related to the anterior temporal region. (b) The scatter plots of the median channel-wise correlation for each chromophore in respect to the [$O_{2}\mathrm{Hb}$] to [HHb] correlation. Red curves indicate the quadratic fit and the orange lines the 95% confidence interval. On the left superior corner, corresponding ρ and $R^{2}$ values for each plot are shown.

We calculated the channel-wise median correlation for each chromophore over all subjects [Fig. 5(a)] and then computed the median value over channels as a metric of global influence. The latter has been plotted against the [$O_{2}\mathrm{Hb}$] to [HHb] correlation results obtained in Sec. 3.2.1 [Fig. 5(b)].

Particularly for [$O_{2}\mathrm{Hb}$] and [tHb], the channels on the anterior temporal region presented a relatively low channel-wise correlation in comparison to the results obtained for all other channels. This can be evidenced from the contrast of colors in the depicted images, which can represent a lower influence of global spontaneous fluctuations on these channels’ signals.

The latter observation is confirmed with the scatter plots showing that channels with stronger [$O_{2}\mathrm{Hb}$] to [HHb] correlation also present a lower influence of global components. While global components seem to be most prominent for [$O_{2}\mathrm{Hb}$] and [tHb], all chromophores presented a strong negative correlation (lower than −0.6) between the global component metric (y-axis) and the [$O_{2}\mathrm{Hb}$] to [HHb] correlation. Also, [$O_{2}\mathrm{Hb}$] and [tHb] presented the highest coefficient of determination ($R^{2}$) for the quadratic curve fit (0.88 and 0.90, respectively).

## 4 Discussion and Conclusions

In this study, we observed consistent positive correlation between [$O_{2}\mathrm{Hb}$] and [HHb] both for task-evoked responses and resting-state data in channels placed over the anterior temporal area of the human head. The positive [$O_{2}\mathrm{Hb}$] to [HHb] correlation phenomenon has been previously suggested to occur due to (i) extracerebral and/or systemic signals,^13^ (ii) motion artifacts and bad coupling,^37^ (iii) cross talk between chromophores,^8^^,^^38^ (iv) predominance of capillaries contribution to the measured hemodynamic signal,^39^ or (v) hemodynamic and oxygenation in muscles.^21^ In the following paragraphs, we will critically discuss the possible contributions of each one of these factors for the interpretation of the findings we observed.

Based on the methods we applied to analyze the data, we believe that our results are likely not due to motion artifacts. The Spearman’s correlation is more robust in the presence of outliers (spike artifacts) in the data and, in addition to that, we have calculated the median correlation over blocks to also increase the robustness to outliers. Also, our results suggest that bad coupling could not explain our findings either, as the illustrative block average signals for the task-related data appear rather clean and there also appears to be a smooth transition of responses patterns while approaching the anterior temporal area. For the resting-state data, we have controlled the coefficient of variation to exclude any channels with potential bad coupling prior to the analysis.

Cross talk between [$O_{2}\mathrm{Hb}$] and [HHb] chromophores has been suggested to occur either when the wavelengths pairs that are used to measure the intensity data and estimate the concentration changes are not optimal^8^ or when the DPF values used for the modified Beer–Lambert law are not precise.^38^ This concern, however, does not apply as the pairs that are employed for NIRx NIRScout (760 and 850 nm) and Hitachi ETG-4000 (695 and 830 nm) are pairs that yield optimal measures.^8^^,^^40^ Also, while for the task-related dataset the concentration changes were based on unknown pathlength factor, for the resting-state data we used a general equation ^29^ to estimate the optimal DPF based on both the wavelength as well as the mean subjects’ age from this study, therefore, we do not expect this to yield cross talk either. Although a dependence of the DPF on anatomical variability is expected,^41^ the large number of subjects examined makes it unlikely this would be the cause of a significant bias.

Finally, the study suggesting a theoretical model to explain the increase of [HHb] as a task-evoked response in regions with a greater density of capillaries in comparison to large vessels^39^ had expected a different spatial localization for the positive [$O_{2}\mathrm{Hb}$] to [HHb] correlation, namely the Broca area. We believe this spatial localization difference may be due to individual anatomical differences, as their study only measured a single subject and considering that the Broca area (inferior frontal gyrus) is very close to the anterior temporal region. However, as their theory does not expect the positive correlation to be present over the temporal region, in our opinion, it could not explain our findings either.

**Fig. 6:**
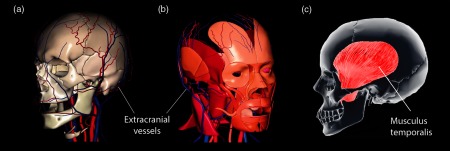
Modified from Ref. 42: extracranial vessels (a) and head muscles (b) of a right-hand specimen. Subfigure (c) highlights the temporalis muscle.

In face of the considerations presented and discussed above, we believe that the spatially localized positive [$O_{2}\mathrm{Hb}$] to [HHb] correlation observed in our study might be better explained by extracerebral changes and/or muscle oxygenation. Figure 6 shows potential anatomical peculiarities concerning the anterior temporal region: a major presence of extracranial vessels and head muscles.

The impact of the extracranial vessels on the measured hemodynamic signals has been previously demonstrated^19^^,^^43^ and this could support the origins of the observed response in our study based on the higher density of superficial vessels as shown in Fig. 6(a). Nevertheless, we consider that the temporalis muscle might yield the most prominent influence on the signals measured over the anterior temporal region because of an intrinsic muscle activity and corresponding hemodynamic change, as it has been demonstrated, e.g., measuring the anterior tibial artery flow with fMRI,^44^ and the vastus lateralis muscle desaturation^45^ as well as resting and exercising skeletal muscles metabolism with NIRS.^46^

It was also recently shown that teeth clenching, which is expected to trigger the activation of temporalis muscle and related hemodynamic changes, yields a pattern of concurrent [$O_{2}\mathrm{Hb}$] and [HHb] increase in channels positioned over the anterior temporal area.^21^ Their report of positive [$O_{2}\mathrm{Hb}$] to [HHb] correlation following particular head muscles activity might be further evidence of the potential source of our task-related findings over the anterior temporal region. Also, in resting-state data, the activation of the temporalis muscle can occur during spontaneous saliva swallowing,^47^ although its frequency is relatively low (1.32  swallows/min).^48^ Additionally, a tonic activation of the temporalis muscle during resting state might be involved in maintaining the mandibular rest position to compensate the gravitational forces pulling the mandible down, as observed by Yilmaz et al.^49^

In addition to the positive [$O_{2}\mathrm{Hb}$] to [HHb] correlation, we explored other metrics in the present study that provide supporting evidence to particularities in the anterior temporal region. With the task-evoked responses, we have observed a close correlation of the observed positive [$O_{2}\mathrm{Hb}$] to [HHb] correlation with the BVPA. This suggests that the simultaneous increase of both [$O_{2}\mathrm{Hb}$] and [HHb] in that area presents non-neuronal origins, as it has been shown that the scalp blood flow exhibits a strong blood volume pulsation corresponding to the heart rate.^50^ Also, we observed that the spontaneous resting-state global fluctuations of both [$O_{2}\mathrm{Hb}$] and [tHb] are not as prominent in the anterior temporal region channels as in all other cortical areas over the left hemisphere, which further supports the idea that there should be a localized source of extracortical signals impacting the measures over the particular anterior temporal area.

Nevertheless, our study presents a few limitations that should be addressed. The first is related to the mean population age assessed with both datasets: 26.0 years for the task-related and 24.2 years for the resting-state data, both with low standard deviation. Therefore, generalization of present findings to other populations, e.g., infants or elderly people, should be done carefully, as an age dependency of the skeletal muscles’ BOLD signal was previously reported.^51^ The second limitation concerns the absence of direct measures of muscle activity (e.g., concurrent electromyography) to be correlated with the hemodynamic changes measured in both mental arithmetic task and resting state. The last limitation is related to the absence of short-distance measurement channels to separate the extracortical information from the cortical signals to assess our theory for the observed findings concerning the temporal extracortical layers.

We conclude that the examination of individual components of hemoglobin signal alone can lead to misinterpretation of fNIRS findings because influence of physiological actions may differ depending on which element is considered. The report of results based on both $O_{2}\mathrm{Hb}$ and HHb is, therefore, important for proper interpretation of anterior temporal-related outcomes. Inclusion of short-distance measurement channels into this region to separate neuronal signals from extracortical components may further improve results interpretation. These guidelines seem to be important not only for studies focused on task-evoked responses, but also for resting-state studies (e.g., connectivity analysis).

## References

[1] Scholkmann F. et al., “A review on continuous wave functional near-infrared spectroscopy and imaging instrumentation and methodology,” NeuroImage 85(1), 6–27 (2014). 10.1016/j.neuroimage.2013.05.004 23684868

[2] Wolf M. Ferrari M. Quaresima V., “Progress of near-infrared spectroscopy and topography for brain and muscle clinical applications,” J. Biomed. Opt. 12(6), 062104 (2007). JBOPFO 1083-3668 10.1117/1.2804899 18163807

[3] Huneau C. Benali H. Chabriat H., “Investigating human neurovascular coupling using functional neuroimaging: a critical review of dynamic models,” Front. Neurosci. 9, 467 (2015). 10.3389/fnins.2015.00467 26733782 PMC4683196

[4] Bale G. Elwell C. E. Tachtsidis I., “From Jöbsis to the present day: a review of clinical near-infrared spectroscopy measurements of cerebral cytochrome-c-oxidase,” J. Biomed. Opt. 21(9), 091307 (2016). JBOPFO 1083-3668 10.1117/1.JBO.21.9.091307 27170072

[5] Kocsis L. Herman P. Eke A., “The modified Beer–Lambert law revisited,” Phys. Med. Biol. 51(5), N91–N98 (2006). PHMBA7 0031-9155 10.1088/0031-9155/51/5/N02 16481677

[6] Yamashita Y. Maki A. Koizumi H., “Wavelength dependence of the precision of noninvasive optical measurement of oxy-, deoxy-, and total-hemoglobin concentration,” Med. Phys. 28(6), 1108–1114 (2001). MPHYA6 0094-2405 10.1118/1.1373401 11439480

[r7] Strangman G. Franceschini M. A. Boas D. A., “Factors affecting the accuracy of near-infrared spectroscopy concentration calculations for focal changes in oxygenation parameters,” NeuroImage 18(4), 865–879 (2003). NEIMEF 1053-8119 10.1016/S1053-8119(03)00021-1 12725763

[8] Uludağ K. et al., “Separability and cross talk: optimizing dual wavelength combinations for near-infrared spectroscopy of the adult head,” NeuroImage 22(2), 583–589 (2004). NEIMEF 1053-8119 10.1016/j.neuroimage.2004.02.023 15193586

[9] Wylie G. R. et al., “Using co-variations in the Hb signal to detect visual activation: a near infrared spectroscopic imaging study,” NeuroImage 47(2), 473–481 (2009). NEIMEF 1053-8119 10.1016/j.neuroimage.2009.04.056 19398013 PMC7201385

[r10] Yoshino K. Kato T., “Vector-based phase classification of initial dips during word listening using near-infrared spectroscopy,” Neuroreport 23(16), 947–951 (2012). NERPEZ 0959-4965 10.1097/WNR.0b013e328359833b 22989928

[11] Hong K.-S. Naseer N., “Reduction of delay in detecting initial dips from functional near-infrared spectroscopy signals using vector-based phase analysis,” Int. J. Neural Syst. 26(3), 1650012 (2016). IJSZEG 0129-0657 10.1142/S012906571650012X 26971785

[12] Villringer A., “Non-invasive optical spectroscopy and imaging of human brain function,” Trends Neurosci. 20(10), 435–442 (1997). 10.1016/S0166-2236(97)01132-6 9347608

[13] Obrig H. Villringer A., “Beyond the visible—imaging the human brain with light,” J. Cereb. Blood Flow Metab. 23(1), 1–18 (2003). 10.1097/01.WCB.0000043472.45775.29 12500086

[14] Caldwell M. et al., “Modelling confounding effects from extracerebral contamination and systemic factors on functional near-infrared spectroscopy,” NeuroImage 143, 91–105 (2016). NEIMEF 1053-8119 10.1016/j.neuroimage.2016.08.058 27591921 PMC5139986

[15] Tachtsidis I. Scholkmann F., “False positives and false negatives in functional near-infrared spectroscopy: issues, challenges, and the way forward,” Neurophotonics 3(3), 030401 (2016). 10.1117/1.NPh.3.3.030401 27054143 PMC4791590

[16] Scholkmann F. et al., “End-tidal ${\mathrm{CO}}_{2}$: an important parameter for a correct interpretation in functional brain studies using speech tasks,” NeuroImage 66, 71–79 (2013). NEIMEF 1053-8119 10.1016/j.neuroimage.2012.10.025 23099101

[17] Scholkmann F. Wolf M. Wolf U., “The effect of inner speech on arterial CO2 and cerebral hemodynamics and oxygenation: a functional NIRS study,” Oxygen Transp. Tissue XXXV 789, 81–87 (2013). 10.1007/978-1-4614-7411-1_12 23852480

[18] Tachtsidis I. et al., “Investigation of frontal cortex, motor cortex and systemic haemodynamic changes during anagram solving,” Oxygen Transp. Tissue XXIX 614, 21–28 (2008). 10.1007/978-0-387-74911-2_3 18290310

[19] Kirilina E. et al., “The physiological origin of task-evoked systemic artefacts in functional near infrared spectroscopy,” NeuroImage 61(1), 70–81 (2012). NEIMEF 1053-8119 10.1016/j.neuroimage.2012.02.074 22426347 PMC3348501

[20] Holper L. Scholkmann F. Wolf M., “The relationship between sympathetic nervous activity and cerebral hemodynamics and oxygenation: a study using skin conductance measurement and functional near-infrared spectroscopy,” Behav. Brain Res. 270, 95–107 (2014). BBREDI 0166-4328 10.1016/j.bbr.2014.04.056 24845305

[21] Volkening N. et al., “Characterizing the influence of muscle activity in fNIRS brain activation measurements,” IFAC–PapersOnLine 49(11), 84–88 (2016). 10.1016/j.ifacol.2016.08.013

[22] Pfurtscheller G. et al., “Focal frontal (de)oxyhemoglobin responses during simple arithmetic,” Int. J. Psychophysiol. 76(3), 186–192 (2010). IJPSEE 0167-8760 10.1016/j.ijpsycho.2010.03.013 20381546

[23] Bauernfeind G., Using Functional Near-Infrared Spectroscopy (fNIRS) for Optical Brain-Computer Interface (oBCI) Applications, PhD Thesis, Graz University of Technology (2012).

[24] “Data sets—BNCI Horizon 2020,” http://bnci-horizon-2020.eu/database/data-sets (6 3 2017).

[25] Jurcak V. Tsuzuki D. Dan I., “10/20, 10/10, and 10/5 systems revisited: their validity as relative head-surface-based positioning systems,” NeuroImage 34(4), 1600–1611 (2007). NEIMEF 1053-8119 10.1016/j.neuroimage.2006.09.024 17207640

[26] Tak S. et al., “Sensor space group analysis for fNIRS data,” J. Neurosci. Methods 264, 103–112 (2016). JNMEDT 0165-0270 10.1016/j.jneumeth.2016.03.003 26952847 PMC4840017

[27] “Supplementary material–NPh 17044SS,” ResearchGate, https://www.researchgate.net/publication/317688424_Supplementary_Material_-_NPh_17044SS (20 6 2017).

[28] Duncan A. et al., “Measurement of cranial optical path length as a function of age using phase resolved near infrared spectroscopy,” Pediatr. Res. 39(5), 889–894 (1996). PEREBL 0031-3998 10.1203/00006450-199605000-00025 8726247

[29] Scholkmann F. Wolf M., “General equation for the differential pathlength factor of the frontal human head depending on wavelength and age,” J. Biomed. Opt. 18(10), 105004 (2013). JBOPFO 1083-3668 10.1117/1.JBO.18.10.105004 24121731

[30] Holper L. Seifritz E. Scholkmann F., “Short-term pulse rate variability is better characterized by functional near-infrared spectroscopy than by photoplethysmography,” J. Biomed. Opt. 21(9), 091308 (2016). JBOPFO 1083-3668 10.1117/1.JBO.21.9.091308 27185106

[31] Trajkovic I. Scholkmann F. Wolf M., “Estimating and validating the interbeat intervals of the heart using near-infrared spectroscopy on the human forehead,” J. Biomed. Opt. 16(8), 087002 (2011). JBOPFO 1083-3668 10.1117/1.3606560 21895329

[32] Scholkmann F. Boss J. Wolf M., “An efficient algorithm for automatic peak detection in noisy periodic and quasi-periodic signals,” Algorithms 5(4), 588–603 (2012).1748-7188 10.3390/a5040588

[33] Obrig H. et al., “Spontaneous low frequency oscillations of cerebral hemodynamics and metabolism in human adults,” NeuroImage 12(6), 623–639 (2000). NEIMEF 1053-8119 10.1006/nimg.2000.0657 11112395

[34] Kleiser S. et al., “Characterizing fluctuations of arterial and cerebral tissue oxygenation in preterm neonates by means of data analysis techniques for nonlinear dynamical systems,” Oxygen Transp. Tissue XXXVII 876, 511–519 (2016). 10.1007/978-1-4939-3023-4_64 PMC6125790 26782252

[35] Carbonell F. Bellec P. Shmuel A., “Global and system-specific resting-state fMRI fluctuations are uncorrelated: principal component analysis reveals anti-correlated networks,” Brain Connect. 1(6), 496–510 (2011). 10.1089/brain.2011.0065 22444074 PMC3604782

[36] Novi S. L. Rodrigues R. B. M. L. Mesquita R. C., “Resting state connectivity patterns with near-infrared spectroscopy data of the whole head,” Biomed. Opt. Express 7(7), 2524–2537 (2016). BOEICL 2156-7085 10.1364/BOE.7.002524 27446687 PMC4948611

[37] Cui X. Bray S. Reiss A. L., “Functional near infrared spectroscopy (NIRS) signal improvement based on negative correlation between oxygenated and deoxygenated hemoglobin dynamics,” NeuroImage 49(4), 3039–3046 (2010). NEIMEF 1053-8119 10.1016/j.neuroimage.2009.11.050 19945536 PMC2818571

[38] Boas D. A. Dale A. M. Franceschini M. A., “Diffuse optical imaging of brain activation: approaches to optimizing image sensitivity, resolution, and accuracy,” NeuroImage 23(Suppl. 1), S275–S288 (2004). NEIMEF 1053-8119 10.1016/j.neuroimage.2004.07.011 15501097

[39] Yamamoto T. Kato T., “Paradoxical correlation between signal in functional magnetic resonance imaging and deoxygenated haemoglobin content in capillaries: a new theoretical explanation,” Phys. Med. Biol. 47(7), 1121–1141 (2002). PHMBA7 0031-9155 10.1088/0031-9155/47/7/309 11996059

[40] Okui N. Okada E., “Wavelength dependence of crosstalk in dual-wavelength measurement of oxy- and deoxy-hemoglobin,” J. Biomed. Opt. 10(1), 011015 (2005). JBOPFO 1083-3668 10.1117/1.1846076 15847581

[41] Ferrari M. et al., “Variability of human brain and muscle optical pathlength in different experimental conditions,” Proc. SPIE 1888, 466 (1993). 10.1117/12.154666

[42] Nowinski W. L. et al., “Three-dimensional stereotactic atlas of the extracranial vasculature correlated with the intracranial vasculature, cranial nerves, skull and muscles,” Neuroradiol. J. 28(2), 190–197 (2015). 10.1177/1971400915576669 25923683 PMC4757164

[43] Takahashi T. et al., “Influence of skin blood flow on near-infrared spectroscopy signals measured on the forehead during a verbal fluency task,” NeuroImage 57(3), 991–1002 (2011). NEIMEF 1053-8119 10.1016/j.neuroimage.2011.05.012 21600294

[44] Towse T. F. et al., “Quantitative analysis of the postcontractile blood-oxygenation-level-dependent (BOLD) effect in skeletal muscle,” J. Appl. Physiol. 111(1), 27–39 (2011). 10.1152/japplphysiol.01054.2009 21330621 PMC3137544

[45] Cettolo V. et al., “Vastus lateralis O2 desaturation in response to fast and short maximal contraction,” Med. Sci. Sports Exercise 39(11), 1949–1959 (2007). 10.1249/mss.0b013e3181453476 17986902

[46] Ferrari M. Binzoni T. Quaresima V., “Oxidative metabolism in muscle,” Philos. Trans. R. Soc. B 352(1354), 677–683 (1997). 10.1098/rstb.1997.0049 PMC1691965 9232855

[47] Monaco A. et al., “Surface electromyography pattern of human swallowing,” BMC Oral Health 8, 6 (2008). 10.1186/1472-6831-8-6 18366770 PMC2294114

[48] Afkari S., “Measuring frequency of spontaneous swallowing,” Australas. Phys. Eng. Sci. Med. 30(4), 313–317 (2007). AUPMDI 0158-9938 18274071

[49] Yilmaz G. et al., “Tonic activity of the human temporalis muscle at mandibular rest position,” Arch. Oral Biol. 60(11), 1645–1649 (2015). AOBIAR 0003-9969 10.1016/j.archoralbio.2015.08.013 26351747

[50] Takahashi T. Takikawa Y. Kawagoe R., “Differences in the pulsatile component of the skin hemodynamic response to verbal fluency tasks in the forehead and the fingertip,” Sci. Rep. 6, 20978 (2016). SRCEC3 2045-2322 10.1038/srep20978 26905432 PMC4764919

[51] Jacobi B. et al., “Skeletal muscle BOLD MRI: from underlying physiological concepts to its usefulness in clinical conditions,” J. Magn. Reson. Imaging 35(6), 1253–1265 (2012). 10.1002/jmri.23536 22588992

